# Going green on wheels: what drives electric vehicle use among self-driving tourists

**DOI:** 10.3389/fpsyg.2026.1839831

**Published:** 2026-06-24

**Authors:** Zhenzhen Xu, Xingbao Hu, Changwei Hu, Mengqian Wu, Hanwen Xing

**Affiliations:** 1 Faculty of International Tourism and Management, City University of Macau, Macau, China; 2 School of Business, Henan University of Science and Technology, Luoyang, China; 3 School of Tourism, Huangshan University, Huangshan, China

**Keywords:** electric vehicle, range anxiety, self-driving tourism, theory of planned behavior, tourists’ intention to use EV

## Abstract

**Introduction:**

Electric vehicles (EVs) play an increasingly important role in supporting the tourism industry’s low-carbon transition. However, the factors and psychological mechanisms underlying tourists’ adoption of EVs for self-driving tourism remain insufficiently understood. Grounded in the Theory of Planned Behavior (TPB), this study develops and validates an extended TPB model to examine how tourist-related factors, EV attributes, and destination conditions jointly influence tourists’ intention to use EVs for self-driving tourism.

**Methods:**

A questionnaire survey was conducted among 545 residents from six representative Chinese cities. Partial least squares structural equation modeling (PLS-SEM) was employed to test the proposed relationships, and PLS multigroup analysis (PLS-MGA) was used to examine gender differences.

**Results:**

The findings indicate that tourists’ intention to use EVs for self-driving tourism is shaped by both individual and contextual factors. Attitude, subjective norms, and perceived behavioral control significantly enhance adoption intention. Price value influences intention indirectly through attitude, whereas limited driving range, charger availability, and policy incentives exert their effects through a perceived behavioral control–range anxiety mechanism. Low-carbon awareness indirectly affects intention through attitude and subjective norms, while range anxiety serves as a key mediator linking perceived behavioral control to intention. Furthermore, gender differences emerge, with male tourists being more strongly influenced by attitude and female tourists being more strongly influenced by subjective norms.

**Discussion:**

This study advances understanding of EV adoption in tourism by revealing the multilevel drivers and psychological mechanisms underlying tourists’ behavioral intentions. It extends the application of TPB to the context of EV-based self-driving tourism and offers practical implications for destination managers, policymakers, and EV developers seeking to promote sustainable mobility in tourism.

## 1 Introduction

Self-driving tourism accounts for more than 40% of tourism activities and continues to grow rapidly, making it a significant segment of the global tourism industry (Au et al., 2024). However, its carbon emissions account for approximately 50% of total transportation-sector emissions (Au et al., 2024), posing serious challenges to both global climate change and destination environmental quality (Tong et al., 2025). The adoption of electric vehicles (EVs) in self-driving tourism is widely regarded as a key pathway toward the low-carbon transition of tourism transportation (Hu et al., 2025). Due to their environmental friendliness, energy efficiency, and lower greenhouse gas emissions, EVs are recognized as a sustainable alternative to conventional gasoline-powered vehicles (Han et al., 2025). Consequently, using EVs for self-driving tourism is emerging as an important future trend in travel and mobility, fundamentally reshaping tourists’ self-driving tourism patterns and experiences (Prideaux, 2020).

Tourist behavior is characterized by spatial concentration, temporal flexibility, and destination-oriented travel patterns (e.g., visiting attractions), whereas consumer behavior tends to involve spatial dispersion, time-regulated, and daily needs-driven activities (e.g., daily life). Consequently, significant differences exist between the two in terms of travel demand and behavioral patterns (Ferreira et al., 2020). However, existing EV research has primarily focused on the transportation sector, particularly on factors influencing consumer commuting and EV purchase behavior (Liao et al., 2017; Jiang et al., 2023; Mesquita et al., 2025). In contrast, studies adopting a tourist perspective remain limited, resulting in insufficient attention to EV-related consumption behavior in tourism contexts (Hu et al., 2025).

Only a few studies have identified the individual factors of tourists, EVs, or destinations that influence using EVs for self-driving tourism, such as destination EV supply and charging infrastructure (Zorn and Suni, 2019), travel habits and prior experiences (Nikiforiadis et al., 2022), EV color design (Au et al., 2024), and hotel EV charger availability (Han et al., 2025; Hu et al., 2025). However, these factors are not entirely independent (Ye et al., 2021), a systematic integration of EV-related, tourist-related, and destination-related factors remains lacking, leading to an insufficient understanding of the driving mechanisms underlying tourists’ intention to use EV. Additionally, existing research has reported significant gender differences in environmental attitudes and behaviors (Echavarren, 2023), which extend to EV usage behaviors (Ma et al., 2025; Xu et al., 2025). However, whether the influence of these factors on tourists’ EV usage intention varies by gender remains unclear.

The theory of planned behavior (TPB) is used to explain the process of individual decision-making and has been widely used to predict pro-environmental and tourism-related behaviors (Ulker-Demirel and Ciftci, 2020). However, existing TPB research has primarily focused on consumers’ EV purchase or daily usage behaviors, while its applicability to the specific context of using EVs for self-driving tourism remains insufficiently examined. Moreover, the TPB’s core variables are limited in their ability to explain behavioral intention, particularly when the external situational factors are not fully accounted for. As a result, in many existing studies, a model’s explanatory power is increased through the incorporation of external variables (Rozenkowska, 2023). This study considers EV-related attributes and destination-related factors as key external antecedents and systematically examines how they influence tourists’ intention to use EV for self-driving tourism through the core constructs of the TPB.

Noteworthily, EVs are generally categorized into battery electric vehicles (BEVs) and hybrid electric vehicles (HEVs) (Schiaroli and Fraccascia, 2026). Unlike HEVs, which combine electric propulsion with conventional fuel engines, BEVs rely entirely on battery-powered electricity. In self-driving tourism contexts, tourists often face long-distance travel, destination uncertainty, and substantial dependence on charging accessibility, making issues such as limited driving range, charging convenience, and range anxiety particularly salient for BEV use. Therefore, consistent with prior literature (Thwe et al., 2025; Schiaroli and Fraccascia, 2026), the term “EV” in this study refers to battery electric vehicles (BEVs). Specifically, this research seeks to address the following three questions:

(1) How do EV factors (e.g., price value, limited driving range, etc.), destination factors (e.g., charger availability, policy incentives, etc.), and tourist factors (e.g., personal mindset, low-carbon awareness, etc.) influence the intention of self-driving tourists to use EVs?
(2) Is there a mediating or chain effect among these three factor types (EV, destination, and tourist factors)?
(3) Do these influence mechanisms differ by tourist gender?

This study offers several key contributions. First, it develops an extension and contextual refinement of the theory of planned behavior (TPB) by integrating tourist-related factors, EV attributes, and destination conditions. In doing so, it broadens the application of TPB in tourism transportation research and clarifies how external contextual factors shape behavioral intention through multiple psychological pathways, including attitude, subjective norms, perceived behavioral control, and range anxiety. Second, this study advances the literature by identifying both the antecedents and behavioral consequences of range anxiety in self-driving tourism. It also reveals significant gender heterogeneity in intention formation: male tourists’ attitudes exert a stronger influence on intention, whereas female tourists are more strongly influenced by subjective norms. Finally, the findings offer actionable implications for destination managers seeking to promote EV-based self-driving tourism and for automakers aiming to optimize EV products and service strategies for tourism markets.

## 2 Literature review and hypothesis development

### 2.1 Electric vehicles and self-driving tourism

Self-driving tourism refers to a form of travel in which individuals use privately owned, rented, or borrowed vehicles as their primary mode of transportation (Zorn and Suni, 2019). It is a crucial segment of the tourism industry in many countries (Prideaux, 2020), accounting for more than 40% of global tourism activity, and it has a sustained growth trend (Au et al., 2024). However, self-driving tourism is responsible for approximately 50% of the carbon emissions derived from transportation, which poses significant challenges to global climate change and the environmental quality of tourist destinations (Au et al., 2024; Tong et al., 2025). Promoting environmentally friendly transportation is key to reducing emissions in tourism travel and achieving sustainable development. Among these strategies, the large-scale adoption of EVs is considered an effective approach (Hu et al., 2025). EVs are widely recognized as green alternatives to gasoline-powered cars (Li et al., 2025) and are prioritized by several countries as an aspect of their strategies for energy conservation, environmental protection, and climate change mitigation (Zhang et al., 2024). Owing to their relatively low operating costs and associations with innovation, energy efficiency, and modern trends, EVs are increasingly being adopted by tourists for self-driving tourism (Fitt, 2022). EV Self-driving tourism is emerging as a key trend in the future of travel (Prideaux, 2020). Therefore, understanding the factors that influence tourists’ willingness to use EVs and the mechanisms underlying this behavior is crucial for promoting the low-carbon transformation of tourism transportation.

Numerous scholars in the transportation field have analyzed the factors that influence ordinary consumers’ decisions to purchase and adopt EVs. These factors can be categorized into three principal areas: (1) The first is personal factors. From an economic perspective, these include gender, age, income, and education level (Liao et al., 2017; Xu et al., 2025). From a psychological standpoint, factors such as attitude, subjective norms, and perceived behavioral control are important (Ye et al., 2021; Thwe et al., 2025; Schiaroli and Fraccascia, 2026). Additionally, concerns such as range anxiety (Rainieri et al., 2023; Ma et al., 2025) and low-carbon awareness (Yang et al., 2022; Xu et al., 2025) also play significant roles. (2) The next area is EV factors. These include the purchase price, price value, charging time, range, safety, convenience, brand, etc. (Liao et al., 2017; Jiang et al., 2023; Mesquita et al., 2025). (3) The third area is social factors. The key factors here include government subsidies, access to preferential policies (Ye et al., 2021; Zhang et al., 2024), charger availability (Szumska and Jurecki, 2021; Vafaei-Zadeh et al., 2022; Mesquita et al., 2025), and the interaction between sellers and customers (Li et al., 2023).

Current research on EV tourism is focused primarily on daily and habitual travel in urban or rural settings (Langbroek et al., 2018; Kester et al., 2020), the design of smart EV charging station recommendation systems in sustainable smart tourism cities (Suanpang et al., 2023), and the business model for the shared use of EVs by tourists at Disney parks (Lesteven and Leurent, 2016). In studies examining the influencing factors, the availability of EVs for rent at tourist destinations, the number of charging facilities (Zorn and Suni, 2019), tourists’ local travel habits, their previous experiences with shared EVs (Nikiforiadis et al., 2022), and even the color design of EVs (Au et al., 2024) significantly impact tourists’ satisfaction with self-driving tourism, their willingness to use shared EVs, and their rental decisions. Additionally, hotel-provided EV charging facilities and their pricing strategies have been shown to significantly increase hotel bookings, willingness to pay, and levels of customer satisfaction (Hu et al., 2025; Li et al., 2025).

Overall, the existing research on the factors that influence EV usage behavior are focused primarily on ordinary consumers (Jiang et al., 2023; Mesquita et al., 2025). However, tourists’ travel behavior differs significantly from that of regular consumers (Ferreira et al., 2020), and studies conducted from the tourist perspective are relatively limited, with insufficient attention given to EV consumption behavior in tourism contexts (Hu et al., 2025). Additionally, research in transportation studies on consumer EV usage behavior has typically focused on individual, EV-related, or social factors. However, these factors do not operate independently (Ye et al., 2021). Moreover, studies that integrate these factors into a single theoretical framework to examine their interactions remain scarce. While some studies have begun to explore the use of EVs in tourism settings, they are often focused on one-dimensional factors. There is still a lack of understanding regarding the influence of multiple factors on tourists’ intention to use EV for self-driving tourism and the underlying psychological mechanisms.

### 2.2 Theory of planned behavior

The theory of planned behavior (TPB) suggests that attitudes, subjective norms, and perceived behavioral control form the foundation of behavioral intention, which in turn influences the formation of specific behaviors (Ajzen, 1991). TPB is a primary model for understanding consumer intention and behavior; it is widely applied in tourism, leisure, and hospitality studies (Ulker-Demirel and Ciftci, 2020) and is the most commonly used theory to predict pro-environmental transportation choices (Jiang et al., 2023). While the TPB has been employed to analyze consumer behavior regarding EV usage in the transportation sector (Thwe et al., 2025; Xu et al., 2025; Schiaroli and Fraccascia, 2026), its application in the context of self-driving tourists using EVs remains limited.

Additionally, the TPB overlooks the impact of external factors, and its variables are insufficient for fully addressing research questions in specific contexts. As a result, previous studies have often enhanced the model by incorporating additional variables or combining it with other theoretical frameworks to improve its predictive power (Ulker-Demirel and Ciftci, 2020; Rozenkowska, 2023). Personal, EV, and social factors are the key determinants of consumers’ EV purchase and usage behavior (Jiang et al., 2023; Mesquita et al., 2025). Thus, the aim of this study is to develop an integrated theoretical model that incorporates both external factors, such as EV attributes and the social environment, and personal psychological processes, such as TPB variables and range anxiety, into the analytical framework to systematically explore the mechanisms influencing tourists’ intention to use EV in self-driving tourism.

### 2.3 Tourist-related factors

#### 2.3.1 Attitude and intention to use EV

According to the TPB, attitude is a reflection of an individual’s positive or negative evaluation of a behavior and serves as a key factor influencing behavioral intention (Ajzen, 1991). Numerous studies have confirmed that the more positive an individual’s attitude toward a behavior is, the more likely he or she is to engage in that behavior (Rozenkowska, 2023). Specifically, attitudes toward low-carbon travel have a significantly positive influence on the intention to engage in low-carbon travel behaviors (Wang et al., 2022; Wang et al., 2023). On this basis, when tourists hold a positive attitude toward using EV for self-driving tourism, they are more likely to develop a stronger intention to use EV for self-driving tourism. Therefore, we propose the following hypothesis:

*H1*: Attitude positively influences self-driving tourists' intention to use EV.

#### 2.3.2 Subjective norms and intention to use EV

Subjective norms represent the social pressure that is perceived during individual behavioral decision-making and are among the antecedent variables that influence behavioral intention in the TPB (Ajzen, 1991). Research shows that the stronger the perceived social pressure is, the greater the behavioral intention (Wang et al., 2023). In the context of tourism, tourists’ behavioral intention toward products and services are also influenced by subjective norms, as tourists are more likely to engage in sustainable behaviors that align with social expectations (Wasaya et al., 2022). Therefore, when tourists perceive greater social pressure regarding the use of EVs for self-driving tourism, they are more likely to develop stronger behavioral intention. Thus, the following hypothesis is formulated:

*H2*: Subjective norms positively influence self-driving tourists' intention to use EV.

#### 2.3.3 Perceived behavioral control, range anxiety, and intention to use EV

Perceived behavioral control refers to an individual’s perception and belief regarding the ease or difficulty of performing a specific behavior (Ajzen, 1991). The strength of perceived behavioral control depends on an individual’s assessment of their own internal capabilities (e.g., skills, abilities) and external conditions (e.g., resources, opportunities). When individuals believe that they possess the necessary abilities and resources to perform a behavior, their perceived behavioral control strengthens, increasing their likelihood of forming corresponding behavioral intention (Thwe et al., 2025). Research has shown that the stronger an individual’s perceived behavioral control regarding green products is, the greater their intention to use them (Vafaei-Zadeh et al., 2022). Similarly, if tourists believe that they have the necessary abilities and resources to use EVs for self-driving tourism, their perceived behavioral control becomes stronger, and they have a greater inclination to use EV for self-driving tourism. On this basis, we propose the following hypothesis:

*H3*: Perceived behavioral control positively influences self-driving tourists' intention to use EV.

Range anxiety refers to the emotional response arising from drivers’ concerns that an EV’s battery capacity and available charging infrastructure may be insufficient to reach their destination (Habla et al., 2021). When tourists purchase products that have inherent risks, concerns about potential negative outcomes may induce anxiety, which can further reduce their behavioral intention as anxiety intensifies (Allen et al., 2019). Existing studies have shown that range anxiety decreases perceived ease of use, driving enjoyment, and perceived safety, thereby negatively influencing EV purchase and usage behavior (Rainieri et al., 2023; Ma et al., 2025).

According to Cognitive Appraisal Theory, individuals first perceive external events in their environment and then evaluate whether they possess sufficient resources and capabilities to cope with them. This appraisal process elicits specific emotional responses, which subsequently shape coping behaviors, forming a “stimulus–evaluation-emotion-response” pathway (Lazarus, 1991). This theoretical perspective helps explain the mediating mechanism underlying tourists’ intention to use EV for self-driving tourism. In the context of EV self-driving tourism, objective constraints such as limited driving range and insufficient charging infrastructure act as external stimuli. Tourists then assess whether they have adequate capabilities and resources to manage these constraints, thereby forming perceptions of behavioral control. Prior studies have shown that perceived lack of control significantly increases anxiety levels (Korte et al., 2015). In contrast, higher perceived behavioral control enhances tourists’ confidence in coping with uncertainties related to EV range and charging, thereby reducing range anxiety during EV use (Rainieri et al., 2023). Therefore, we propose that perceived behavioral control indirectly enhances participation intention by alleviating range anxiety.

*H4*: Range anxiety negatively affects self-driving tourists' intention to use EV.

*H5*: Range anxiety mediates the relationship between perceived behavioral control and self-driving tourists' intention to use EV.

#### 2.3.4 Low-carbon awareness and intention to use EV

Low-carbon awareness refers to the values, attitudes, and knowledge that promote environmental protection through the reduction of greenhouse gas emissions (Du et al., 2018). Environmental concerns are a significant influencing factor on tourists’ intention to use EV (Mesquita et al., 2025). Although EVs face technological and range limitations, consumers and tourists with strong low-carbon awareness are more accepting of EVs and other low-carbon products because of their energy-saving and environmentally friendly characteristics, which leads to more positive attitudes toward their use (Jia et al., 2018). Additionally, Value-Attitude-Behavior (VAB) theory suggests that values influence attitudes, which in turn shape behavior, leading to causal progression (Homer and Kahle, 1988). Thus, low-carbon awareness can enhance tourists’ positive attitudes toward using EVs for self-driving tourism, which in turn strengthens their behavioral intention. The following hypothesis thus applies:

*H6*: Attitude mediates the relationship between low-carbon awareness and self-driving tourists' intention to use EV.

The stronger that an individual’s environmental awareness is, the greater their perception of society’s emphasis and expectations on environmental protection, which increases the social pressure they feel and strengthens their subjective norms in regard to environmental behaviors (Yang et al., 2022). These subjective norms make individuals more likely to engage in pro-environmental behaviors, such as the purchase of electric vehicles, to align with societal expectations (Xu et al., 2025). Existing research has confirmed that subjective norms mediate the relationship between low-carbon awareness and low-carbon travel intention (Liu et al., 2017). Therefore, this study posits that tourists’ low-carbon awareness enhances their perception of social expectations regarding using EVs for self-driving tourism, thereby strengthening their subjective norms and ultimately increasing their intention to use EV for self-driving tourism. We propose the following hypothesis:

*H7*: Subjective norms mediate the relationship between low-carbon awareness and self-driving tourists' intention to use EV.

### 2.4 EV-related factors

#### 2.4.1 Price value and intention to use EV

Price value refers to the utility that consumers derive from perceived cost savings for the use of a product (Sweeney and Soutar, 2001). According to the theory of consumer perceived value, perceived value refers to consumers’ overall evaluation of utility based on the trade-off between perceived benefits and perceived sacrifices. It encompasses multiple dimensions, including quality, price, emotional, and social value, all of which influence consumers’ attitudes and behaviors (Sweeney and Soutar, 2001; Zhang et al., 2020). Electricity costs are lower than the costs of fossil fuels (Habla et al., 2021), and EVs have fewer moving parts than traditional vehicles, resulting in lower maintenance costs (Liu et al., 2021). When tourists perceive lower energy consumption and maintenance costs associated with using EVs for self-driving tourism, they develop a higher perceived price value. This positive evaluation of cost-effectiveness leads to more favorable attitudes toward using EVs for self-driving tourism (Vafaei-Zadeh et al., 2022). According to TPB, such positive attitudes further increase tourists’ intention to use EV for self-driving tourism. Existing studies have also shown that, in the context of energy-saving appliances, price value influences consumers’ willingness to pay a price premium through the mediating role of attitudes (Zhang et al., 2020).

*H8*: Attitude mediates the relationship between price value and self-driving tourists' intention to use EV.

#### 2.4.2 Limited driving range and intention to use EV

EVs are limited by battery capacity and energy consumption rates, resulting in a lower range than that of traditional gasoline vehicles, with the range potentially decreasing by up to 50% during cold temperatures (Steinstraeter et al., 2021). This limited range has become a key factor that objectively constrains tourists’ intention to use EVs for self-driving tourism (Fitt, 2022). Perceived behavioral control involves an individual’s assessment of his or her abilities and control over external resources (Ajzen, 2002). Range limitation, as an external factor beyond personal control, diminishes tourists’ confidence and sense of control in completing trips during using EVs for self-driving tourism, which lowers their perceived behavioral control and weakens their behavioral intention.

Additionally, according to the “stimulus–evaluation-emotion-response” pathway from Cognitive Appraisal Theory (Lazarus, 1991), when tourists encounter range limitations as an external stimulus, their perceived behavioral control decreases, which increases their range anxiety and ultimately affects their intention to use EVs for self-driving tourism. On this basis, we propose the following hypotheses:

*H9*: Perceived behavioral control and range anxiety sequentially mediate the relationship between limited driving range and self-driving tourists' intention to use EV.

### 2.5 Destination-related factors

Social support theory posits that social support, which includes instrumental support such as material and informational resources, can foster positive cognitive and behavioral outcomes for individuals (Chouhy et al., 2020). Both facilities and policy support are integral components of destination support (Chen et al., 2023). Among these forms of support, charging stations serve as essential infrastructure for EVs (Vafaei-Zadeh et al., 2022). However, compared with traditional fueling stations, the current number of EV charging stations is insufficient, which is a key barrier that prevents tourists from using EVs for self-driving tourism (Szumska and Jurecki, 2021). Additionally, to mitigate carbon emissions, several countries have implemented policies aimed at promoting the adoption of EVs (Jiang et al., 2023). Destination incentive policies facilitate operations and mitigate safety risks, serving as a critical institutional driver of using EVs for self-driving tourism (Spence, 1978). Perceived behavioral control, which reflects a combination of self-efficacy and the perceived level of control over external factors, influences behavioral intention (Ajzen, 2002). Accordingly, sufficient charging facilities and strong policy support can increase tourists’ sense of safety and confidence in EV self-driving tourism, thereby increasing their perceived behavioral control (Chen et al., 2023).

According to the “stimulus–evaluation-emotion-response” progression model proposed in Cognitive Appraisal Theory (Lazarus, 1991), when tourists perceive higher levels of destination infrastructure quality and policy support, their confidence in the use of EVs for self-driving tourism increases (Chen et al., 2023). This increased confidence reduces such emotional responses as range anxiety, ultimately enhancing self-driving tourists’ intention to use EV for self-driving tourism. Therefore, the following hypotheses are proposed:

*H10*: Perceived behavioral control and range anxiety serially mediate the relationship between charger availability and self-driving tourists' intention to use EV.

*H11*: Perceived behavioral control and range anxiety serially mediate the relationship between policy incentive and self-driving tourists' intention to use EV.

### 2.6 The impact of tourist gender

According to socialization theory, women are often socialized into roles that emphasize care, altruism, and nurturing, whereas men are typically socialized to prioritize independence and competition. This socialization discrepancy results in women generally having higher levels of environmental awareness and engaging in more pro-environmental behaviors than men do (Echavarren, 2023). Additionally, research has indicated that gender differences significantly influence individuals’ decision-making tendencies in terms of risk perception, technology acceptance, and travel preferences (Sovacool et al., 2019). Therefore, gender, as a key social characteristic, may play a crucial role in shaping tourists’ decision-making processes regarding EV adoption in self-driving tourism. In the context of transportation and tourism, men and women often demonstrate different behavioral patterns in their adoption of new technologies, formation of environmental attitudes, and selection of travel modes (Zhang et al., 2022; Ma et al., 2025; Xu et al., 2025). Drawing on these insights, this study advances the hypothesis that the factors influencing self-driving tourists’ intention to use EV may differ systematically between genders.

*H12*: The effects of tourist factors, EV factors, and destination factors on self-driving tourists' intention to use EV vary between the genders.

**Figure 1:**
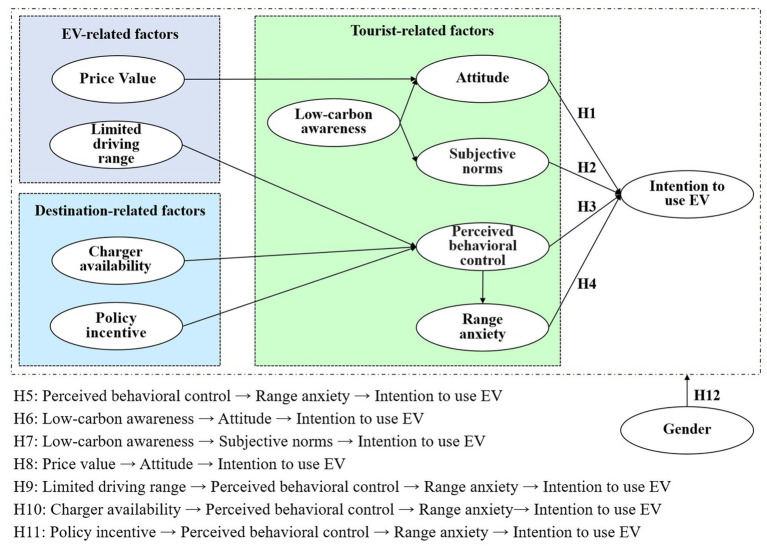
Research model.

Based on the above hypotheses, this study develops a theoretical model of tourists’ EV use intention for self-driving tourism (see Figure 1).

## 3 Methodology

### 3.1 Construct measurement and data collection

All the construct measures used in this study were adapted from established scales in the literature, with minor modifications made to fit the research context (see Appendix 1). The attitude items were adapted from Ajzen (2002). The measures of subjective norms, perceived behavioral control, and intention to use EV were drawn from Han (2015). Charger availability and price value were based on Vafaei-Zadeh et al. (2022), respectively. Limited driving range and policy incentive items were adapted from the scales developed by Franke and Krems (2013) and Chen et al. (2025). Range anxiety was measured using items adapted from Rauh et al. (2020), whereas low-carbon awareness was assessed using four items from Jia et al. (2018). All items were measured on a five-point Likert scale, with responses ranging from 1 = “strongly disagree” to 5 = “strongly agree.”

Data were collected using a time-based systematic sampling approach, in which respondents were selected at fixed time intervals. This method improves sample representativeness and has been previously applied in tourism research (Wu and Lai, 2025). Data collection was conducted in two phases, from March to April 2024 and in early May 2026, across six Chinese cities: Guangzhou, Shenzhen, Beijing, Chengdu, Wuhan, and Hangzhou. China has become the world’s largest EV market (Zhang et al., 2024), and self-driving tourism has become a dominant travel mode among Chinese tourists (Tong et al., 2025), new energy vehicles are expected to become a mainstream mode of transport for self-driving tourism (People's Daily Online, 2024). Moreover, a growing body of research on EV adoption and green travel behavior has employed Chinese samples for empirical investigation (e.g., Li et al., 2023; Chen et al., 2025; Ma et al., 2025; Ma et al., 2026). These cities were selected because they are national EV promotion demonstration cities with well-developed EV sales networks and supporting infrastructure. In addition, potential consumers in these cities generally possess substantial awareness of and exposure to EVs, enabling the collection of high-quality sample data (Li et al., 2023). Data were collected in the parking areas of popular natural and cultural attractions across these cities. Trained research assistants invited one self-driving tourist every 20 min. After having the study purpose explained and giving their informed consent, the respondents were invited to complete a questionnaire. All respondents were screened using qualifying questions to ensure that they had no prior experience with EV self-driving tourism but possessed some knowledge of EVs.

A total of 751 questionnaires were distributed. After the removal of incomplete and invalid responses, 545 valid questionnaires were retained. This sample size exceeds the minimum requirement of 168 as calculated using the inverse square root method (Kock and Hadaya, 2016), thus indicating sufficient statistical power. Table 1 presents the respondents’ demographic profile. Males accounted for 54.3% of the sample, slightly exceeding females (45.7%). Most respondents were aged 25–54 years (78.1%), and 73.8% held a bachelor’s degree or above. Regarding monthly income, the largest group reported earning CNY 6,001–10,000 per month (26.6%). In terms of travel frequency, 41.1% reported taking 2–4 trips per year.

**Table 1:** Demographic characteristics of respondents.

Item	Characteristic	Frequency	Percentage (%)
Gender	Male	296	54.3
	Female	249	45.7
Age	18–24	82	15.0
	25–34	116	21.3
	35–44	197	36.1
	45–54	113	20.7
	≥55	37	6.8
Education	Junior Middle school or below	27	5.0
	Junior college	116	21.3
	Bachelor’s degree	298	54.7
	Master’s degree or above	104	19.1
Monthly personal income (CNY)	3,000 or less	85	15.6
	3,001-6,000	101	18.5
	6,001-10,000	145	26.6
	10,001-20,000	116	21.3
	≥20,001	98	18
Average annual travel frequency	Once or less	162	29.7
	2–4 times	224	41.1
	5–7 times	103	18.9
	8 times or more	56	10.3

### 3.2 Analysis methods

Partial least squares structural equation modeling (PLS-SEM) was employed to test the proposed research model. This method is well suited for exploratory and predictive research and is particularly effective for analyzing complex theoretical models involving mediating and sequential mediation effects (Hair et al., 2019). PLS-SEM has also been widely applied in hospitality and tourism research (Usakli and Kucukergin, 2018). The analyses were conducted using SmartPLS 4.0 and followed a three-step procedure. First, the reliability and validity of the measurement model were assessed to establish a sound basis for hypothesis testing. Second, the structural model was evaluated by examining the path coefficients and assessing their statistical significance to test the proposed theoretical relationships. Third, PLS multigroup analysis was performed to examine the moderating role of gender, thereby identifying boundary conditions in the formation of self-driving tourists’ behavioral intention.

## 4 Analysis results

### 4.1 Measurement model assessment

During data collection, several procedural remedies were implemented to minimize common method bias (CMB), including anonymous responses, emphasizing that there were no right or wrong answers, randomizing and separating questionnaire items, and adopting well-established measurement scales. In addition, Harman’s single-factor test was conducted to assess CMB, and the first factor accounted for only 32.044% of the variance (Podsakoff et al., 2003), indicating that the data were free from common method bias. Furthermore, the latent common method factor approach was applied to further examine CMB (Williams et al., 2003; Liang et al., 2007). The results showed that the average substantively explained variance of the indicators was 0.773, while the average method-based variance was only 0.0007. The substantial difference between the two ratios (602:1, see Appendix 2), together with the finding that most method factor loadings were statistically insignificant, further suggested that CMB did not pose a significant threat to the study (Liang et al., 2007). Moreover, all the values of the variance inflation factor (VIF) between the constructs were less than 3.3 (ranging from 1.000 to 1.616), suggesting that there was no multicollinearity (Kock, 2015).

As shown in Table 2, all the factor loadings exceeded the threshold of 0.7 (Hair et al., 2019), affirming the indicators’ reliability. The Cronbach’s alpha (CA) and composite reliability (CR) values also surpassed the threshold of 0.7, indicating reliability in internal consistency (Hair et al., 2019). Additionally, all of the average variance extracted (AVE) values were above 0.5, which demonstrates satisfactory convergent validity (Hair et al., 2019).

**Table 2:** Assessment of measurement model.

Construct/item	Loading	Cronbach’s α	CR	AVE
Attitude (AT)		0.908	0.909	0.785
AT1	0.917			
AT2	0.857			
AT3	0.895			
AT4	0.873			
Charger availability (CA)		0.827	0.833	0.743
CA1	0.855			
CA2	0.837			
CA3	0.893			
Intention to use EV (IU)		0.843	0.847	0.761
IU 1	0.899			
IU 2	0.875			
IU 3	0.842			
Low-carbon awareness (LA)		0.889	0.889	0.750
LA1	0.860			
LA2	0.863			
LA3	0.864			
LA4	0.878			
Limited driving range (LDR)		0.869	0.870	0.792
LDR1	0.901			
LDR2	0.881			
LDR3	0.888			
Perceived behavioral control (PBC)		0.854	0.859	0.773
PBC1	0.895			
PBC2	0.857			
PBC3	0.885			
Policy incentive (PI)		0.894	0.901	0.759
PI1	0.880			
PI2	0.896			
PI3	0.863			
PI4	0.844			
Price value (PV)		0.843	0.847	0.760
PV1	0.877			
PV2	0.880			
PV3	0.859			
Range anxiety (RA)		0.920	0.927	0.806
RA1	0.892			
RA2	0.866			
RA3	0.907			
RA4	0.926			
Subjective norms (SN)		0.817	0.817	0.733
SN1	0.870			
SN2	0.867			
SN3	0.831			

CR, Composite Reliability; AVE, Average Variance Extracted.

As Table 3 shows, the square roots of the AVEs were greater than the inter-construct correlations (Hair et al., 2019), and the heterotrait-monotrait ratios (HTMTs) of the correlations were less than the threshold of 0.85 (Hair et al., 2019). Thus, discriminant validity was well established (Hair et al., 2019).

**Table 3:** Discriminant validity.

Construct	(1)	(2)	(3)	(4)	(5)	(6)	(7)	(8)	(9)	(10)
Attitude	**0.886**	0.277	0.522	0.411	0.415	0.547	0.346	0.378	0.224	0.403
Charger availability	0.241	**0.862**	0.490	0.438	0.185	0.378	0.405	0.197	0.217	0.294
Intention to use EV	0.459	0.411	**0.873**	0.534	0.467	0.585	0.676	0.508	0.404	0.588
Low-carbon awareness	0.371	0.376	0.461	**0.866**	0.394	0.488	0.424	0.403	0.223	0.440
Limited driving range	−0.370	−0.157	−0.401	−0.346	**0.890**	0.657	0.375	0.346	0.467	0.347
Perceived behavioral control	0.484	0.319	0.499	0.426	−0.566	**0.879**	0.455	0.466	0.467	0.450
Policy incentive	0.315	0.350	0.590	0.379	−0.333	0.401	**0.871**	0.403	0.216	0.436
Price value	0.332	0.165	0.430	0.349	−0.296	0.396	0.351	**0.872**	0.199	0.358
Range anxiety	−0.205	−0.193	−0.359	−0.204	0.418	−0.419	−0.199	−0.173	**0.898**	0.146
Subjective norms	0.348	0.243	0.488	0.375	−0.292	0.376	0.374	0.298	−0.127	**0.856**

The bold values on the diagonal are the square roots of the AVE. Below diagonal, inter-construct correlations; above diagonal, HTMT ratios.

### 4.2 Structural model assessment

**Figure 2:**
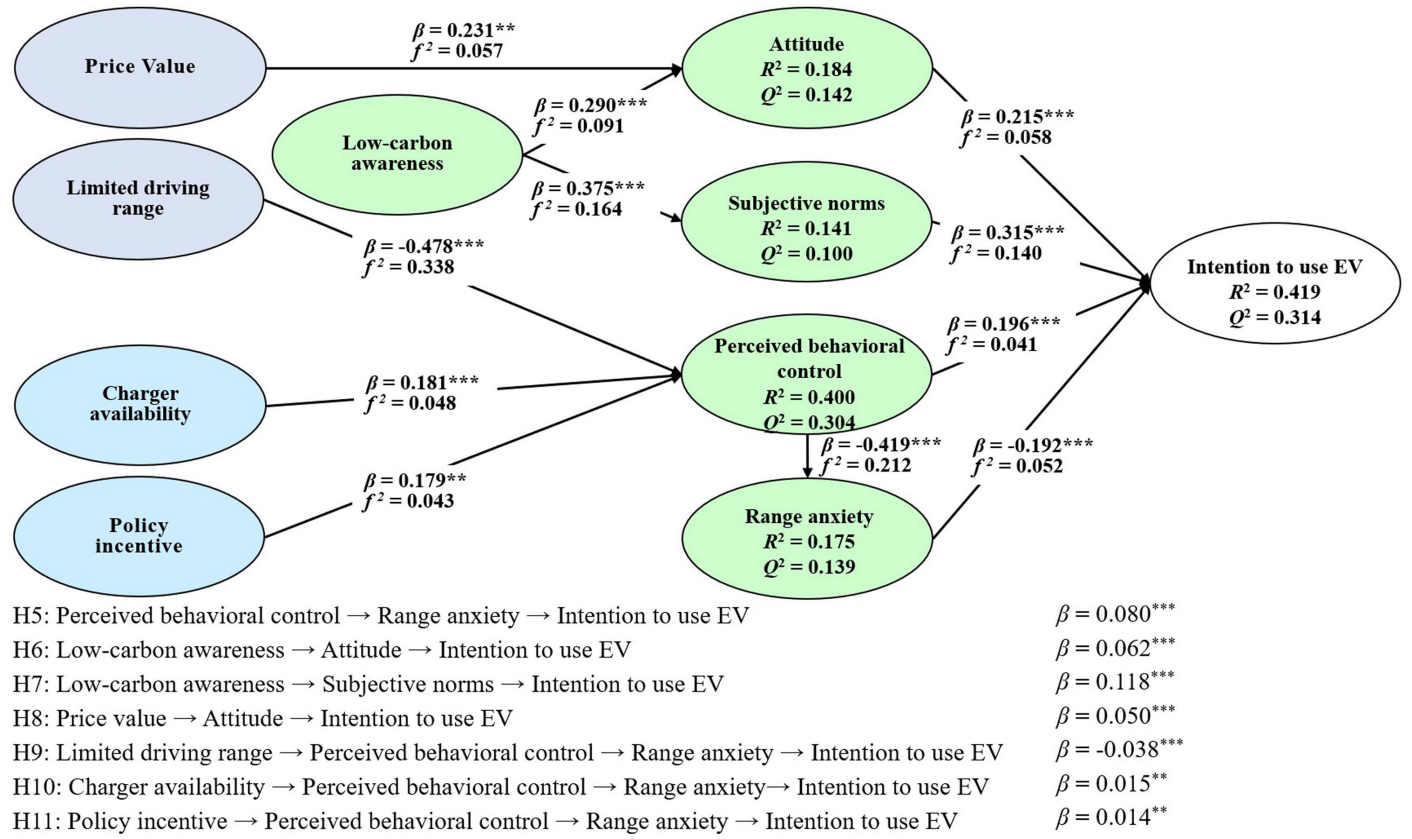
Structural model results.

As shown in Figure 2, the *R*^2^ values were between 0.141 and 0.419, indicating a moderate to strong moderate explanatory power (Cohen, 2013). The *Q*^2^ values ranged from 0.100 to 0.314 suggested the model’s adequate predictive relevance (Hair et al., 2019). In addition, and all *f*^2^ values exceeded 0.02, indicating that the *f*^2^ values in this study were acceptable (Hair et al., 2019).

The path coefficients were estimated using 5,000 bootstrap resamples. As shown in Table 4, attitude (*β* = 0.215, *p* < 0.001), subjective norms (*β* = 0.315, *p* < 0.001), and perceived behavioral control (*β* = 0.196, *p* < 0.001) significantly positively affected intention to use EV. In contrast, range anxiety had a significant negative effected on this intention (*β* = −0.192, *p* < 0.001), which supported H1, H2, H3, and H4.

**Table 4:** Structural model results.

Hypothesis	Path	*β*	*t*-value	*p*-value	Result
H1	Attitude → intention to use EV	0.215	5.915	0.000^***^	Supported
H2	Subjective norms → intention to use EV	0.315	8.770	0.000^***^	Supported
H3	Perceived behavioral control → intention to use EV	0.196	4.589	0.000^***^	Supported
H4	Range anxiety → intention to use EV	−0.192	5.031	0.000^***^	Supported
H5	Perceived behavioral control → range anxiety → intention to use EV	0.080	4.693	0.000^***^	Supported
H6	Low-carbon awareness → attitude → intention to use EV	0.062	4.176	0.000^***^	Supported
H7	Low-carbon awareness → subjective norms → intention to use EV	0.118	5.743	0.000^***^	Supported
H8	Price value → attitude → intention to use EV	0.050	3.774	0.000^***^	Supported
H9	Limited driving range → perceived behavioral control → range anxiety → Intention to use EV	−0.038	4.364	0.000^***^	Supported
H10	Charger availability → perceived behavioral control → range anxiety → Intention to use EV	0.015	3.361	0.001^**^	Supported
H11	Policy incentive → perceived behavioral control → range anxiety → intention to use EV	0.014	3.086	0.002^**^	Supported

^**^p < 0.01; ^***^p < 0.001.

Mediation analysis using PLS-SEM revealed that range anxiety mediated the relationship between perceived behavioral control and self-driving tourists’ intention to use EV (*β* = 0.08, *p* < 0.001), supporting H5. Additionally, the findings showed that low-carbon awareness positively influenced tourists’ intention to use EV through attitude (*β* = 0.062, *p* < 0.001) and subjective norms (*β* = 0.118, *p* < 0.001), supporting H6 and H7. Attitude also mediated the relationship between price value and tourists’ intention to use EV (*β* = 0.05, *p* < 0.001), supporting H8. Finally, limited driving range (*β* = −0.038, *p* < 0.001), charger availability (*β* = 0.015, *p* < 0.01), and policy incentive (*β* = 0.014, *p* < 0.01) influenced tourists’ intention to use EV through mediated pathways involving perceived behavioral control and range anxiety, which supported H9, H10, and H11.

### 4.3 Moderating effect of tourist gender

Following the procedure outlined in Henseler et al. (2016) for assessing the measurement invariance of composite models (MICOM), partial measurement invariance was established across different groups (see Appendix 3), which supported the subsequent use of PLS-MGA. As shown in Table 5, significant differences were found between male and female tourists in the paths for H1, H2, and H6. Specifically, for H1, the effect of attitude on tourists’ intention to use EV was significantly stronger for male tourists than it was for female tourists (*Δβ* = 0.241, *p* < 0.01). For H2, the impact of subjective norms on intention to use EV was weaker for male tourists than it was for female tourists (*Δβ* = −0.225, *p* < 0.01). Additionally, for H4, the indirect effect of low-carbon awareness on intention to use EV through attitude was significantly stronger for the male group than it was for the female group (*Δβ* = 0.077, *p* < 0.01), providing partial support for H12.

**Table 5:** PLS-MGA results for tourist gender.

Path	Male		Female		Male vs. female	
	*β*	*p*-value	*β*	*p*-value	△*β*	*p*-value
Attitude → intention to use EV	0.322	0.000	0.081	0.135	0.241	0.001^**^
Subjective norms → intention to use EV	0.204	0.000	0.429	0.000	−0.225	0.001^**^
Perceived behavioral control → intention to use EV	0.242	0.000	0.166	0.007	0.076	0.360
Range anxiety → intention to use EV	−0.145	0.006	−0.222	0.000	0.078	0.320
Perceived behavioral control → range anxiety → intention to use EV	0.065	0.009	0.085	0.001	−0.020	0.577
Low-carbon awareness → attitude → intention to use EV	0.099	0.000	0.022	0.191	0.077	0.008^**^
Low-carbon awareness → subjective norms → intention to use EV	0.084	0.001	0.148	0.000	−0.065	0.130
Price value → attitude → intention to use EV	0.064	0.004	0.023	0.186	0.041	0.129
Limited driving range → perceived behavioral control → range anxiety → intention to use EV	−0.032	0.012	−0.039	0.002	0.007	0.701
Charger availability → perceived behavioral control → range anxiety → intention to use EV	0.015	0.020	0.010	0.091	0.005	0.560
Policy incentive → perceived behavioral control → range anxiety → intention to use EV	0.013	0.048	0.014	0.046	−0.002	0.865

**p < 0.01.

## 5 Conclusion and implications

### 5.1 Main findings and discussion

Grounded in the theory of planned behavior (TPB), this study integrates tourist-, EV-, and destination-related factors to examine the determinants and psychological mechanisms underlying self-driving tourists’ intention to use EVs. The key findings are as follows:

From the perspective of EV- and destination-related factors, price value indirectly influences tourists’ intention to use EV through attitude, which differs from the direct effect on purchase intention reported by Vafaei-Zadeh et al. (2022). A possible explanation is that price, as an economic factor, may directly shape intention in commodity purchase decisions. However, this study focuses on potential adoption of EVs for future self-driving tourism rather than product purchase decisions. For potential EV self-driving tourism adopters, who lack actual EV tourism experience, price information alone may not be sufficient to directly translate into behavioral intention. Instead, tourists may be more concerned with whether EVs can meet their expectations for travel quality and experiential value during self-driving tourism (Fitt, 2022). Therefore, cost-related considerations may first be internalized into attitudinal evaluations before further influencing behavioral intention.

Furthermore, perceived behavioral control and range anxiety sequentially mediate the relationships between limited driving range, charger availability, policy incentive, and tourists’ intention to use EV. This finding closely aligns with the “stimulus–evaluation–emotion–response” sequence proposed in Cognitive Appraisal Theory (Lazarus, 1991) and reveals the underlying cognition–emotion mechanism through which external EV- and destination-related factors shape behavioral intention. In this sense, the study provides empirical support for the applicability of Cognitive Appraisal Theory in the context of using EVs for self-driving tourism. Compared with the relatively stable and predictable range demands associated with daily commuting, using EVs for self-driving tourism typically involves longer travel distances, unfamiliar road environments, and greater uncertainty regarding charging infrastructure availability. Consequently, uncertainty associated with range limitations becomes substantially amplified, making tourists’ perceptions of whether the journey can be successfully managed particularly important. Under such conditions, perceived behavioral control plays a critical buffering role in alleviating range anxiety. This finding suggests that, for tourists without prior EV self-driving tourism experience, range anxiety is not merely a direct consequence of objective technological constraints, but rather a psychologically constructed perception of travel-related risk grounded in tourists’ subjective cognitive evaluations.

From the tourist perspective, the core TPB variables—attitude, subjective norms, and perceived behavioral control—positively influence tourists’ intention to use EV for self-driving tourism. These findings are consistent with prior research on EV purchase intention and adoption behavior (Thwe et al., 2025; Xu et al., 2025) and extend previous findings from vehicle purchase decisions to the context of using EVs for self-driving tourism. Moreover, perceived behavioral control indirectly influences behavioral intention by reducing range anxiety, forming a sequential pathway of “perceived behavioral control → range anxiety → behavioral intention.” For tourists without prior EV self-driving tourism experience, perceived ability to manage and control the trip serves as a key psychological resource for alleviating anxiety associated with adopting an unfamiliar travel mode powered by a different energy source. It also empirically supports Rainieri et al.’s (2023) argument that perceived behavioral control is crucial for mitigating range anxiety and facilitating EV adoption, while extending this mechanism to the self-driving tourism context.

Furthermore, attitude and subjective norms, respectively, mediate the relationship between low-carbon awareness and tourists’ intention to use EV. Consistent with the Value-Attitude-Behavior (VAB) framework (Homer and Kahle, 1988), tourists’ values in hedonic and experience-oriented tourism contexts do not necessarily translate directly into behavioral intention; instead, they exert indirect effects through favorable attitudinal evaluations toward using EVs for self-driving tourism. This finding further extends the applicability of the VAB framework to sustainable tourism mobility contexts. Additionally, the mediating role of subjective norms suggests that, for potential EV self-driving tourism adopters, approval from family members, friends, and the broader social environment can further strengthen the translation of low-carbon awareness into behavioral intention. In this regard, the present study extends Liu et al.’s (2017) findings on low-carbon travel behavior to the EV tourism context. More importantly, previous studies have reported inconsistent findings regarding whether environmental awareness significantly promotes EV adoption intention (Schiaroli and Fraccascia, 2026). The present study offers a possible explanation for these inconsistencies by identifying two distinct mechanisms through which low-carbon awareness influences behavioral intention: the attitudinal pathway and the social normative pathway.

Finally, this study revealed significant gender differences in the formation of EV self-driving tourism adoption intention among tourists without prior EV self-driving tourism experience. Specifically, for male tourists, both the effect of attitude on EV self-driving intention and the indirect effect of low-carbon awareness through attitude were significantly stronger. In contrast, for female tourists, subjective norms exerted a stronger positive influence on behavioral intention. These findings can be interpreted through Social Role Theory and Socialization Theory. According to Social Role Theory, long-standing divisions of social labor have historically associated men with economic provision and independent decision-making, whereas women have been more closely linked to caregiving and relational maintenance (Eagly, 2013). Socialization Theory further suggests that these role expectations become gradually internalized through repeated social interactions, ultimately shaping gender-specific values and behavioral patterns. Such gender differences in environmental attitudes and behaviors have been widely documented in prior research (Echavarren, 2023).

In the context of using EVs for self-driving tourism, male tourists tend to place greater emphasis on personal evaluations, autonomy, and functional judgments, thereby strengthening the influence of attitude on behavioral intention. Female tourists, by contrast, are generally more sensitive to interpersonal relationships and social approval, making them more likely to rely on the opinions of significant others and prevailing social norms when forming travel decisions. While previous studies have primarily focused on gender differences in EV purchase behavior (Zhang et al., 2022; Xu et al., 2025) and driving behavior (Ma et al., 2025), this study extends the discussion to tourism contexts and enriches the theoretical understanding of green travel intention formation from a social psychological perspective. Future research could further examine the role of gender differences in EV self-driving tourism adoption across different cultural and social contexts.

### 5.2 Theoretical implications

First, this study extends the application of the Theory of Planned Behavior (TPB) to the specific context of using EVs for self-driving tourism, thereby enriching its applicability within tourism transportation research. Compared with daily commuting, using EVs for self-driving tourism is characterized by greater spatial uncertainty, tighter temporal constraints, and stronger dependence on energy replenishment infrastructure (Ferreira et al., 2020). Although TPB has been widely employed to explain pro-environmental intention and tourist behavioral mechanisms (Ulker-Demirel and Ciftci, 2020), its applicability to using EVs for self-driving tourism remains underexplored. By integrating EV-related attributes, destination-related factors, and tourist characteristics into the TPB framework, this study provides an extension and contextual refinement of TPB in EV self-driving tourism settings and empirically validates the proposed model. Unlike prior studies that primarily treated external variables as direct antecedents influencing behavioral intention through TPB constructs such as attitude and subjective norms (Vafaei-Zadeh et al., 2022; Thwe et al., 2025; Xu et al., 2025), this study further identifies a chained psychological mechanism of “Perceived Behavioral Control → Range Anxiety → Intention” in using EVs for self-driving tourism. This finding offers a more nuanced explanation of psychological processes in tourism mobility contexts and advances the theoretical application of TPB in using EVs for self-driving tourism.

Furthermore, this study offers novel insights into the decision-making process of tourists planning to use EVs for self-driving tourism, highlighting the dynamic interplay between the external factors (e.g., EV features and destination attributes) and the internal psychological variables (e.g., attitudes, subjective norms, perceived control, and range anxiety). By examining the indirect relationships among these factors, this research deepens our theoretical understanding of tourist behavior in the context of EVs, thereby filling a gap in the literature, which is predominantly focused on EV adoption outside the tourism sector (Hu et al., 2025). Unlike prior studies in which individual factors, such as the EV charging infrastructure (Zorn and Suni, 2019), traveler characteristics (Nikiforiadis et al., 2022), EV design features (Au et al., 2024), and hotel charging services (Hu et al., 2025; Li et al., 2025) have been considered in isolation, this research emphasizes the complex interactions among these various determinants. This discovery transcends previous research that has merely identified “which factors exert influence” by clearly revealing the transmission mechanism of “external factors → internal psychology → behavioral intention.” This fundamentally deepens our understanding of tourist decision-making. Furthermore, this study aligns with the recommendation of Zorn and Suni (2019) to further explore the specific mechanisms through which various factors influence tourists’ EV travel behavior.

Additionally, this study extends the exploration of the antecedents and consequences of range anxiety, which is a critical psychological barrier hindering the widespread adoption of EVs (Ma et al., 2025). While previous research has consistently shown that range anxiety negatively impacts EV usage behavior, the interactions among individual psychological factors, EV characteristics, and societal influences in triggering range anxiety remain unclear (Rainieri et al., 2023). Through empirical analysis, this study introduces a chain mediation model— “EV and destination factors → perceived behavioral control → range anxiety → intention to use EV,” which not only broadens the antecedents of range anxiety from a singular perspective to a systematic view encompassing “EV-Destination-Visitor” but also extends the consequences of range anxiety from EV usage in transportation to the specific behavioral decisions that are made in road trip contexts. Additionally, this study responds to the call made by Fitt (2022) to strengthen the empirical exploration into the impact of range anxiety on the tourism experience. Future studies could explore strategies for enhancing perceived behavioral control through EV performance and destination factors to alleviate range anxiety in EV self-driving tourism.

Lastly, this study reveals gender-based decision-making heterogeneity in EV self-driving tourism, thereby enhancing the theoretical understanding of the differences between the genders in terms of environmental behavior. Existing research has often suggested that women tend to exhibit higher environmental consciousness than men (Echavarren, 2023), resulting in stronger EV-adoption intention among women than among men (Xu et al., 2025). However, our findings reveal that male tourists’ decisions regarding EV self-driving tourism are driven primarily by intrinsic attitudes, whereas those of female tourists are influenced more strongly by external social norms. This insight provides a novel perspective for understanding the intricate relationship between gender and environmental behavior.

### 5.3 Practical implications

The findings offer several practical implications for governments, tourism destinations, and EV providers seeking to promote using EVs for self-driving tourism.

First, destinations should improve both the availability and visibility of EV charging infrastructure. For tourists without prior EV self-driving tourism experience, assessments of the feasibility of EV self-driving tourism are largely based on external information and environmental cues rather than actual usage experience. Therefore, charging stations should be strategically located near major attractions, hotels, and rest areas along tourism routes. In addition, destinations should provide real-time information on charger locations, availability, and connector compatibility through official tourism websites, mobile applications, and mainstream navigation platforms. Digital tools such as route-planning services, predictive range mapping, and charging reservation systems can further help potential EV adopters assess trip feasibility more accurately, thereby strengthening perceived behavioral control and alleviating range anxiety.

Second, governments and destinations should introduce diversified incentive policies, such as discounted attraction tickets, EV-exclusive parking privileges, charging subsidies, priority lane access, and exemptions from traffic restrictions (Jiang et al., 2023). Such measures can strengthen tourists’ positive perceptions of using EVs for self-driving tourism and increase their willingness to participate.

Third, communication strategies should move beyond general low-carbon advocacy and focus on reducing psychological barriers to EV adoption among potential users. Destination managers and EV providers can encourage tourists with prior EV self-driving tourism experience to share authentic travel stories through short videos, travel blogs, and low-carbon tourism campaigns. Such user-generated content can provide non-users with vicarious experiences, enhance their understanding of EV travel, and reduce uncertainty associated with EV use in tourism contexts. By observing the successful experiences of other tourists, potential users may develop more favorable attitudes toward EV self-driving tourism and greater confidence in adopting EVs for future travel. Meanwhile, opinion leaders, peer recommendations, and user-generated content can be leveraged to cultivate supportive social norms, thereby helping translate environmental awareness into behavioral intention.

Additionally, EV providers should strengthen experiential and scenario-based marketing. Rather than relying solely on competitive pricing or subsidies, firms should communicate the value of EVs in tourism contexts to potential EV self-driving tourism adopters through transparent pricing, bundled travel packages, EV experience centers, and digital marketing platforms (Ye et al., 2021). In addition, differentiated communication strategies can be adopted for different tourist groups. For tourist segments more strongly influenced by attitudes, promotional content may emphasize functional value, driving performance, technological advantages, and travel autonomy. For segments more strongly influenced by subjective norms, marketing should highlight user reviews, social recommendations, and group endorsement cues.

### 5.4 Limitations and future research

This study has three key limitations. Firstly, it focuses on Chinese self-driving tourists without prior experience using EVs for self-driving tourism. Cultural, regional, and tourism-context differences may affect the generalizability of the proposed model. Future research could validate the framework across different countries, cultures, and tourism contexts, such as urban and rural tourism settings, and compare potential adopters with actual EV self-driving tourism users. In addition, given that EVs include different types, future research could further explore whether tourists’ perceptions and behavioral mechanisms vary across EV subgroups, thereby providing a more fine-grained understanding of EV-based self-driving tourism. Finally, because this study focuses on potential adopters rather than current EV self-driving tourism users, it does not further distinguish how tourists intend to access EVs for future self-driving trips. Future research could compare adoption intentions under privately owned EV and EV rental scenarios to examine whether different access modes lead to distinct adoption decisions.

## Data Availability

The raw data supporting the conclusions of this article will be made available by the authors, without undue reservation.
