# Supplementary material for: Going green on wheels: what drives electric vehicle use among self-driving tourists
Source: Front Psychol. 2026 Jun 24;17:1839831. doi: 10.3389/fpsyg.2026.1839831 (PMC13341804; doi:10.3389/fpsyg.2026.1839831)
Supplement: Supplementary file 1 [file Table_1.docx]

Appendix 1. Construct scales

| Constructs | Items | | Reference |
| --- | --- | --- | --- |
| Attitude (AT) | I believe using EVs for self-driving tourism: | | Ajzen (2002) |
|  | AT1 | is excellent. |  |
|  | AT2 | is an eco-friendly way for travel. |  |
|  | AT3 | is an economical way for travel. |  |
|  | AT4 | is an innovative way for travel. |  |
| Subjective norms (SN) | SN1 | Most people who are important to me think I should use EVs for self-driving travel. | Han (2015) |
|  | SN2 | Most people who are important to me would support my use of EVs for self-driving travel. |  |
|  | SN3 | people whose opinions I value would approve of my use of EVs for self-driving travel. |  |
| Perceived behavioral control  (PBC) | PBC1 | Whether or not to use EVs for self-driving travel is entirely under my control. | Han (2015) |
|  | PBC2 | I am confident in my ability to use EVs for self-driving travel. |  |
|  | PBC3 | I have sufficient resources, time, and opportunities to use EVs for self-driving travel. |  |
| Limited driving range  (LDR) | When using EVs for self-driving tourism, | | Franke and Krems (2013) |
|  | LDR1 | The driving range of EVs does not adequately meet my travel needs. |  |
|  | LDR2 | The limitation of driving range is a barrier to usage. |  |
|  | LDR3 | Compared to conventional gasoline vehicles, the driving range is limited. |  |
| Charger availability  (CA) | CA1 | EV charging stations are sufficiently available along my self-driving travel routes. | Vafaei-Zadeh et al. (2022) |
|  | CA2 | EV charging facilities are available at my travel destination. |  |
|  | CA3 | I can easily locate EV charging stations along major travel routes, such as highways and main roads. |  |
| Price value (PV) | Compared with the self-driving travel using conventional gasoline vehicles, | | Vafaei-Zadeh et al. (2022) |
|  | PV1 | Using EVs for self-driving travel is cost-effective. |  |
|  | PV2 | Using EVs for self-driving travel offers good value for money. |  |
|  | PV3 | Using EVs for self-driving travel is economical in the long term. |  |
| Policy incentive (PI) | PI1 | Tourism destinations provide special subsidies for using EVs in self-driving tourism (e.g., travel vouchers or toll fee exemptions). | Chen et al. (2025) |
|  | PI2 | Tourism destinations offer discounts or subsidies for EV charging fees. |  |
|  | PI3 | Tourism destinations provide dedicated parking spaces or discounted parking fees for EVs. |  |
|  | PI4 | EVs are exempt from vehicle license plate restriction policies. |  |
| Range anxiety (RA) | When using EVs for self-driving tourism, | | Rauh et al. (2020) |
|  | RA1 | I worry that the driving range of the EV may not be sufficient to complete my trip. |  |
|  | RA2 | I feel stressed, anxious, or even fearful about not having enough driving range. |  |
|  | RA3 | I am often troubled by the possibility of getting stranded due to running out of battery. |  |
|  | RA4 | I frequently think about the need to recharge the EV during the trip. |  |
| Low-carbon awareness  (LA) | LA1 | Saving energy and reducing carbon emissions are necessary ways to improve the environment. | Jia et al. (2018) |
|  | LA2 | I believe controlling environmental pollution is an urgent task. |  |
|  | LA3 | I think I should make efforts to protect the environment. |  |
|  | LA4 | I am willing to save energy and reduce carbon emissions in my daily life. |  |
| Intention to use EV (IU) | IU1 | I am willing to use EVs for self-driving travel in the future. | Han (2015) |
|  | IU2 | I plan to choose EVs instead of conventional gasoline vehicles for future self-driving travel. |  |
|  | IU3 | I will use EVs for self-driving travel rather than using a traditional combustion engine car in the future. |  |

Note: Electric Vehicle (EV)

Appendix 2. Results of common latent factor approach

| Indicator | Substantive Factor Loading (R1) | R1^2^ | Method Factor Loading (R2) | R2^2^ |
| --- | --- | --- | --- | --- |
| AT1 | 0.920^***^ | 0.846 | -0.007 | 0.000049 |
| AT2 | 0.847^***^ | 0.717 | 0.060 | 0.0036 |
| AT4 | 0.897^***^ | 0.805 | -0.044 | 0.001936 |
| AT5 | 0.878^***^ | 0.771 | -0.007 | 0.000049 |
| CA1 | 0.855^***^ | 0.731 | 0.023 | 0.000529 |
| CA2 | 0.844^***^ | 0.712 | -0.036 | 0.001296 |
| CA3 | 0.886^***^ | 0.785 | 0.012 | 0.000144 |
| IU1 | 0.896^***^ | 0.803 | 0.035 | 0.001225 |
| IU2 | 0.874^***^ | 0.764 | 0.001 | 0.000001 |
| IU3 | 0.847^***^ | 0.717 | -0.039 | 0.001521 |
| LA1 | 0.860^***^ | 0.740 | 0.019 | 0.000361 |
| LA2 | 0.862^***^ | 0.743 | 0.012 | 0.000144 |
| LA3 | 0.863^***^ | 0.745 | 0.012 | 0.000144 |
| LA4 | 0.879^***^ | 0.773 | -0.043 | 0.001849 |
| LDR1 | 0.899^***^ | 0.808 | -0.030 | 0.0009 |
| LDR2 | 0.881^***^ | 0.776 | -0.002 | 0.000004 |
| LDR3 | 0.890^***^ | 0.792 | 0.032 | 0.001024 |
| PBC1 | 0.890^***^ | 0.792 | 0.058 | 0.003364 |
| PBC2 | 0.865^***^ | 0.748 | -0.082 | 0.006724^*^ |
| PBC3 | 0.883^***^ | 0.780 | 0.020 | 0.0004 |
| PI1 | 0.880^***^ | 0.774 | -0.011 | 0.000121 |
| PI2 | 0.888^***^ | 0.789 | 0.075 | 0.005625^*^ |
| PI3 | 0.864^***^ | 0.746 | 0.002 | 0.000004 |
| PI4 | 0.852^***^ | 0.726 | -0.068 | 0.004624 |
| PV1 | 0.864^***^ | 0.746 | 0.033 | 0.001089 |
| PV2 | 0.892^***^ | 0.796 | -0.009 | 0.000081 |
| PV3 | 0.861^***^ | 0.741 | -0.023 | 0.000529 |
| RA1 | 0.894^***^ | 0.799 | 0.024 | 0.000576 |
| RA2 | 0.872^***^ | 0.760 | 0.039 | 0.001521 |
| RA3 | 0.904^***^ | 0.817 | -0.039 | 0.001521^*^ |
| RA4 | 0.922^***^ | 0.850 | -0.021 | 0.000441 |
| SN1 | 0.873^***^ | 0.762 | -0.017 | 0.000289 |
| SN2 | 0.871^***^ | 0.759 | -0.017 | 0.000289 |
| SN3 | 0.824^***^ | 0.679 | 0.036 | 0.001296 |
| Average | 0.876 | 0.767 | -0.000059 | 0.001273 |

Note: AT = Attitude, IU = Intention to use EV, CAV = Charger availability, LA = Low-carbon awareness, LDR = Limited driving range, PI = Policy incentive, PBC = Perceived behavioral control, RA = Range anxiety, SN = Subjective norms, PV = Price value; *p < 0 .050; **p < 0.01; ***p < 0.001.

Appendix 3. MICOM result

| Construct | Step 1 | Step 2 | | | | Step 3a | | Step 3b | | Full  Measurement Invariance |
| --- | --- | --- | --- | --- | --- | --- | --- | --- | --- | --- |
|  | Configurational Invariance | Original correlation | 5%  quantile | Permutation *p*-value | Compositional invariance | Mean-original difference | Conﬁdence  Interval | Mean-original difference | Conﬁdence  Interval |  |
| AT | Yes | 1.000 | 0.999 | 0.653 | Yes | 0.006 | [-0.169,0.162] | 0.192 | [-0.193 ,0.204] | Yes / Yes |
| CA | Yes | 0.998 | 0.993 | 0.444 | Yes | -0.065 | [-0.174,0.175] | 0.092 | [-0.181,0.193] | Yes / Yes |
| IU | Yes | 1.000 | 0.999 | 0.704 | Yes | 0.070 | [-0.160,0.165] | 0.025 | [-0.184,0.179] | Yes / Yes |
| LA | Yes | 0.999 | 0.998 | 0.248 | Yes | 0.021 | [-0.161,0.180] | 0.131 | [-0.247,0.236] | Yes / Yes |
| LDR | Yes | 1.000 | 0.999 | 0.951 | Yes | 0.054 | [-0.167,0.158] | -0.020 | [-0.128,0.126] | Yes / Yes |
| PBC | Yes | 1.000 | 0.999 | 0.933 | Yes | 0.010 | [-0.167,0.166] | 0.014 | [-0.186,0.175] | Yes / Yes |
| PI | Yes | 1.000 | 0.998 | 0.893 | Yes | -0.033 | [-0.162,0.178] | -0.010 | [-0.204,0.209] | Yes / Yes |
| PV | Yes | 0.999 | 0.994 | 0.751 | Yes | -0.035 | [-0.164,0.162] | -0.013 | [-0.211,0.205] | Yes / Yes |
| RA | Yes | 1.000 | 0.999 | 0.268 | Yes | 0.179 | [-0.166,0.169] | 0.049 | [-0.132 ,0.123] | NO / Yes |
| SN | Yes | 1.000 | 0.997 | 0.715 | Yes | 0.295 | [-0.159,0.169] | -0.104 | [-0.232,0.213] | NO / Yes |

Note: AT = Attitude, IU = Intention to use EV, CAV = Charger availability, LA = Low-carbon awareness, LDR = Limited driving range, PI = Policy incentive, PBC = Perceived behavioral control, RA = Range anxiety, SN = Subjective norms, PV = Price value; **p* < 0 .050; ***p* < 0.01; ****p* < 0.001.
